# Unusual Effect of Subcutaneous Injection

**Published:** 1868-07

**Authors:** F. Woodhouse Braine


### Unusual Effect of Subcutaneous Injection.

BY F. WOODHOUSE BRAINE, F. R. C. S., ETC.

Mrs. H. C., aged 35, in good health otherwise, had been kept awake for seventy-two hours by intense neuralgic pain on left side of head, face, and neck, arising from a carious molar tooth on the left side of lower jaw. She was injected with morphacet—1-3 gr. At 1 A. M. on June 28th last, the morphis, dissolved in about four drops of water, was introduced under the skin of the left arm, just over the insertion of the deltoid. No blood appeared at the puncture. In about fifteen seconds, tightness of the chest and difficulty in breathing was complained of, and the patient asked to be raised, saying she felt as if she were dying. Her face and lips now became pale; speech became indistinct (not audible;) pulse irregular; some spasm of the facial muscles took place, and she fell back to all appearance dead. Cold water was freely dashed over face and chest, and, as she was unable to swallow, her tongue was rubbed over with sal volatile, and ammonia rubbed to her nose, artificial respiration being kept up at the same time. During this time her face was blanched, pulse not to be felt, and respiration not to be perceived. Insensibility continued for about three minutes; then happily, one or two feeble beats of the pulse, and a shallow inspiration or two, showed returning animation. She then became conscious; pulse feeble but regular; respiration slow; fingers remained numbed, and both thumbs were firmly drawn into the palms of the hands. This passed off in about six minutes, leaving her feeling very ill, but free from the neuralgic pain, which did not return. There was no feeling of nausea and no attempt at vomiting during any part of the time *Medical Times and Gazette.—Monthly Medical Reprint.*

